# Early therapeutic effects of adaptive servo-ventilation on cardiac sympathetic nervous function in patients with heart failure evaluated using a combination of ^11^C-HED PET and ^123^I-MIBG SPECT

**DOI:** 10.1007/s12350-017-1132-4

**Published:** 2017-11-27

**Authors:** Yusuke Tokuda, Mamoru Sakakibara, Keiichiro Yoshinaga, Shiro Yamada, Kiwamu Kamiya, Naoya Asakawa, Takashi Yoshitani, Keiji Noguchi, Osamu Manabe, Nagara Tamaki, Hiroyuki Tsutsui

**Affiliations:** 1 Department of Cardiovascular Medicine, Hokkaido University Graduate School of Medicine, Kita 15, Nishi 7, Kita-ku, Sapporo, 060-8638 Japan; 2 Department of Cardiovascular Medicine, Tokyo Tenshi Hospital, Tokyo, Japan; 3 Diagnostic and Therapeutic Nuclear Medicine, National Institute of Radiological Sciences, 4-9-1 Anagawa, Inage-Ku, Chiba, 263-8555 Japan; 4 Department of Cardiovascular Medicine, Otaru Kyokai Hospital, Otaru, Japan; 5 Department of Cardiovascular Medicine, Hakodate Neurosurgery Hospital, Hakodate, Japan; 6 Department of Nuclear Medicine, Hokkaido University Graduate School of Medicine, Sapporo, Japan; 7 Department of Cardiovascular Medicine, Graduate School of Medical Sciences, Kyushu University, Fukuoka, Japan

**Keywords:** ^11^C-hydroxyephedrine, ^123^I-metaiodobenzylguanidine, adaptive servo-ventilation, cardiac sympathetic nerve function, heart failure, tomography

## Abstract

**Rationale:**

Adaptive servo-ventilation (ASV), a novel respiratory support therapy for sleep disorders, may improve cardiac function in heart failure (HF). However, the reasons that ASV improves cardiac function have not been fully studied especially in sympathetic nervous function (SNF). The purpose of the present study was to investigate the effects of ASV therapy on cardiac SNF in patients with HF.

**Methods:**

We evaluated ASV therapeutic effects before and 6 months after ASV therapy in 9 HF patients [57.3 ± 17.3 years old, left ventricular ejection fraction (LVEF) 36.1 ± 16.7%]. We performed echocardiography, polysomnography, biomarkers, ^11^C-hydroxyephedrine (HED) PET as a presynaptic function marker and planar ^123^I-metaiodobenzylguanidine (MIBG) to evaluate washout rate.

**Results:**

ASV therapy reduced apnea-hypopnea index (AHI) and improved plasma brain natriuretic peptide (BNP) concentration. In ^123^I-MIBG imaging, the early heart/mediastinum (H/M) ratio increased after ASV therapy (2.19 ± 0.58 to 2.40 ± 0.67; *P* = 0.045). Washout rate did not change (23.8 ± 7.3% to 23.8 ± 8.8%; *P* = 0.122). Global ^11^C-HED retention index (RI) improved from 0.068 ± 0.033/s to 0.075 ± 0.034/s (*P* = 0.029).

**Conclusions:**

ASV reduced AHI and improved BNP. ASV might initially improve presynaptic cardiac sympathetic nervous function in HF patients after 6 months of treatment.

**Electronic supplementary material:**

The online version of this article (10.1007/s12350-017-1132-4) contains supplementary material, which is available to authorized users.

## Introduction

Sympathetic nervous function (SNF) is an important compensatory mechanism to maintain cardiovascular homeostasis in chronic heart failure (HF).^1^,^2^ However, continuous activation of cardiac SNF induces myocardial remodeling through increased active oxygen uptake and alters the signal transduction system of the beta-adrenergic receptor, resulting in increased long-term mortality in HF.^3^ Angiotensin-converting enzyme inhibitors,^4^ angiotensin receptor blockers,^5^ beta-blockers,^6^ and aldosterone antagonists^7^ are thought to inhibit myocardial remodeling induced by cardiac SNF, by acting as neurohormonal antagonists, and to improve long-term mortality in patients with HF.

Sleep-related breathing disorders are one of the comorbidities that worsen HF via inducing alterations in loading conditions, hypoxia, and SNF activation.^8^,^9^ Continuous positive airway pressure (CPAP) treatment has been shown to improve cardiac function in patients with HF and obstructive sleep apnea.^8^ Adaptive servo-ventilation (ASV) has also been reported to improve cardiac function and may be a promising non-pharmacological strategy for the treatment of HF.^10^,^11^ However, the effects of ASV on HF patients have not been established.^12^,^13^ A previous study reported that abnormal breathing patterns in HF patients were regulated with ASV by enhancing pulmonary vagal afferents, resulting in suppression of SNF.^14^ Additionally, ASV could convert rapid shallow breathing patterns to slow regular breathing patterns and prevent respiratory oscillations in HF patients, which could also inhibit cardiac SNF.^14^,^15^ Therefore, the evaluation of cardiac SNF is essential to determining the therapeutic mechanisms behind ASV in HF.

^123^I-metaiodobenzylguanidine (^123^I-MIBG) single-photon and ^11^C-hydroxyephedrine positron emission tomography (^11^C-HED PET) have enabled the assessment of cardiac sympathetic nerve function (SNF).^16^,^17^^123^I-MIBG planar imaging is considered a standard approach to evaluating cardiac SNF and predicting prognosis in HF patients.^18^^–^22 The properties of MIBG allow for it to be used to measure not only the heart/mediastinum (H/M) ratio as a presynaptic function marker but also the washout rate as a partial reflection of norepinephrine (NE) turnover parameter of SNF. Koyama et al.^13^ evaluated the therapeutic effects of ASV using ^123^I-MIBG imaging. It is well known that ^123^I-MIBG imaging has insufficient image quality and the accuracy of semi-quantitative parameters is suboptimal. In contrast, ^11^C-HED PET has emerged as a novel modality providing a high-quality image and it can be used to accurately quantitatively evaluate cardiac SNF including the retention index (RI).^17^,^23^,^24^ However, ^11^C-HED PET cannot be used to assess the washout rate because of its short physical half-life.^25^ Using combined ^11^C-HED PET and ^123^I-MIBG imaging, it should be possible to measure precise presynaptic cardiac sympathetic nervous function and sympathetic turnover. Accordingly, we examined the effects of ASV therapy on cardiac SNF using ^11^C-HED PET as a quantitative presynaptic function parameter and ^123^I-MIBG imaging as a partial reflection of NF turnover parameter in patients with HF.

## Methods

### Study Population

Eleven consecutive patients with chronic HF were prospectively enrolled in the current study at Hokkaido University Hospital between August 2010 and August 2013. HF was defined as New York Heart Association function (NYHA) class II or III, left ventricular ejection fraction (LVEF) < 50%, and plasma brain-type natriuretic peptide (BNP) concentration > 100 pg/mL. Major exclusion criteria included uncontrolled decompensated HF, active ischemic heart disease with exertion chest pain and requiring coronary revascularization, severe pulmonary disease, neurological or musculoskeletal disease, severe valvular regurgitation, stroke, respiratory failure, end-stage renal disease treated by hemodialysis, and inability to tolerate ASV. All patients had received optimal medical therapy to stabilize their symptoms and signs of HF before participating in the study.

The study was approved by the Ethics Committee of Hokkaido University Hospital. All participants provided written informed consent.

### Study Design

Patients received nocturnal ASV treatment for 6 months. Pharmacological therapy was not changed during the study period. ASV (AutoSet CS; Teijin Pharma, Tokyo, Japan) was used without oxygenation and administered via a fitted nasal mask (Teijin Pharma) in the default mode, with expiratory positive airway pressure of 5 cmH_2_O and inspiratory positive airway pressure of 3–10 cmH_2_O. We instructed the patients to use ASV > 4 h per night during the study period. Data regarding compliance of ASV and effectiveness were stored in the device, downloaded and analyzed. Adequate adherence to ASV was defined as > 4 hours per night.^13^ We performed ^11^C-HED PET/computed tomography (CT), ^123^I-MIBG imaging, laboratory tests, polysomnography, and echocardiography in all patients before and after 6 months of ASV treatment. All the imaging data obtained before and after ASV therapy were blindly analyzed in order to avoid interpreter bias.

### Blood and Urine Biochemistry

Plasma concentrations of BNP and norepinephrine and the urinary concentration of norepinephrine were measured at baseline and after 6 months of ASV treatment.

### Overnight Polysomnography

Patients underwent overnight polysomnography using a cardiopulmonary monitoring device (Morpheus; Teijin Limited, Tokyo, Japan) comprising a pressure sensor for nasal flow, two stress-sensitive belts for the ribcage and abdomen, and a continuous pulse oximeter.^26^,^27^

### Echocardiography

We measured left ventricle (LV) end-diastolic dimension, LV end-systolic dimension, LVEF, interventricular septal thickness, LV posterior wall thickness, and the ratio between early and late diastolic transmitral flow velocity using echocardiography (Aplio Artida SSH-880-CV or Aplio SSA-770A; Toshiba Medical Systems, Tochigi, Japan). We also measured the ratio of maximal early diastolic filling wave velocity to maximal early diastolic myocardial velocity, and deceleration time. LVEF was measured from apical four- and two-chamber images using the biplane method of disks.

### ^123^I-MIBG Imaging

Patients received 111 MBq of ^123^MIBG (FUJIFILM RI Pharma, Tokyo, Japan) at rest under fasting conditions.^23^ Fifteen minutes and 4 h after the ^123^MIBG injection, planar images and SPECT data acquisitions were performed using a dual-head scanner (Millennium MG; Elgems, Tirat Carmel, Israel). Whole cardiac ^123^I-MIBG uptake was measured as the H/M ratio using a planar image. The region of interest (ROI) was manually traced for the whole LV and a rectangular ROI was placed in the upper mediastinum in the early image.^18^ Rectangular ROIs of 9 × 9 pixels were placed in the upper 30% of the lung vertical length. The same ROI was applied for the delayed ^123^I-MIBG planar image. The H/M ratio was calculated by dividing the count density of the whole heart by that of the mediastinum, and standardized using the calibration phantom method.^28^ We calculated the washout ratio using the following equation:

washout rate = ([H-M] at 15 min − [H-M] at 4 h/(H-M) at 15 min × 100,

where H = mean counts per pixel in the left ventricle and M = mean counts per pixel in the upper mediastinum.^29^

Although we obtained ^123^MIBG imaging data with planar and SPECT, we did not use SPECT data for evaluation. We did not apply a physical decay correction for the H/M calculation and the method of washout rate.

### ^11^C-HED PET/CT Imaging

^11^C-HED was produced from [^11^C] methyl iodide and metaraminol (free base) using standard methods with high purity and high specific activity. Patients were instructed to fast overnight. ^11^C-HED PET/CT imaging was performed with a 64-slice PET/CT scanner (Biograph Siemens/CTI, Knoxville, TN). Low-dose CT was also performed for attenuation correction. CT co-registered standard orthogonal PET images were re-sliced into series of short-axis, horizontal long-axis, and vertical long-axis images.

Immediately after intravenous administration of 5 mCi (185 MBq) ^11^C-HED, 40-min three-dimensional list-mode PET acquisition was performed.^30^ Images were reconstructed using filtered back correction with a 12-mm Hann filter and were reconstructed into 23 frames (10 × 10, 1 × 60, 5 × 100, 3 × 180, and 4 × 300 seconds).^23^ During analysis, regional ^11^C-HED PET/CT uptake was assessed using the 17-segment model recommended by the American Society of Nuclear Cardiology. Then, the LV wall was subdivided into four segments: anterior, septum, inferior, and lateral wall. The ^11^C-HED RI was calculated as

$$ {\text{Retention}}\;{\text{index}} = \frac{{{\text{Tissue}}\;{\text{activity}}\left( {30 - 40\;{ \hbox{min} }} \right)}}{{\mathop \smallint \nolimits_{0}^{40} {\text{blood}}\;{\text{activity}}}} $$

**Figure 1:**
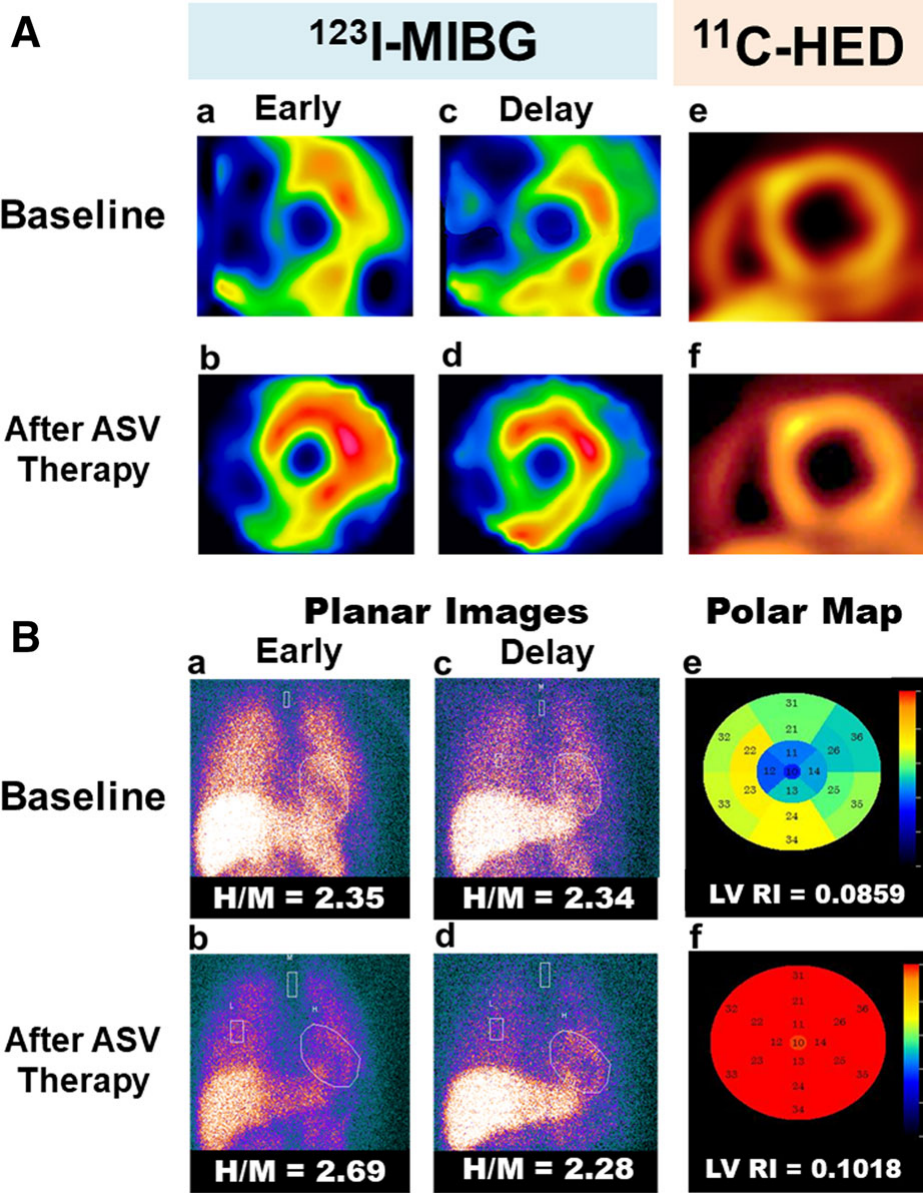
Representative images of 27-year-old female with ^123^I-MIBG planar/SPECT and ^11^C-HED PET/CT. **A** Short-axis images of ^123^I-MIBG SPECT and ^11^C-HED PET/CT. **B** Planar images of ^123^I-MIBG and polar map images of ^11^C-HED PET/CT. **a** Early ^123^I-MIBG SPECT images at baseline. **b**^123^I-MIBG SPECT images after 6 months of ASV. **c** Delayed ^123^I-MIBG SPECT images at baseline. **d** Delayed ^123^I-MIBG SPECT images after 6 months of ASV. **e**^11^C-HED PET/CT images at baseline. **f**^11^C-HED PET images after 6 months of ASV. Representative case of 27-year-old female with heart failure with improved AHI (15.6 to 0.5/h) and LVEF (38.0 to 45.0%) after ASV therapy. Global ^11^C-HED PET/CT RI, a presynaptic function marker of SNF, significantly improved after ASV treatment (Figure 1B-e, 1B-e at baseline; and 1B-f after ASV). The early H/M ratio also increased (Figure 1B-a at baseline; and 1B-b after ASV), whereas the delayed H/M ratio, a turnover parameter of SNF, did not change (Figure 1B-c at baseline; and 1B-d after ASV). *ASV*, adaptive servo-ventilation; *HED*, ^11^C-hydroxyephedrine; ^*123*^*I-MIBG SPECT*, ^123^I-metaiodobenzylguanidine single-photon emission computed tomography; *PET*, positron emission tomography; *H/M*, heart/mediastinum; *RI*, retention index; *SNF*, sympathetic nervous function; *LVEF*, left ventricular ejection fraction

This equation yields a value for the RI based on mean tracer counts within the myocardial ROI between 30 and 40 min divided by the integrated metabolite-corrected time-plasma radioactivity curve from 0 to 40 min after injection.^24^,^31^ Mean RI of all slices was taken as the measure of ^11^C-HED retention. The LV wall was divided into 17 segments based on ASNC guidelines using in-house software^30^,^32^. Then, we divided these 17 segments into four territories (anterior, septum, inferior, and lateral wall) and calculated the mean value in each region (Figure 1).^23^

### Statistical Analysis

Values are mean ± standard deviation. Categorical variables are presented as frequencies with percentages. Since BNP levels showed non-linearity, plasma BNP levels were calculated using log-transformation.^30^ We analyzed data distribution using Shapiro-Wilk tests. Continuous variables with normal distribution at baseline and after ASV treatment were compared using the paired t-test. In case of non-normal distribution, we applied the Wilcoxon signed-rank test to evaluate the statistical differences between measurements at baseline and 6 months after the ASV therapy. A *P* value < 0.05 was considered statistically significant. All analyses were performed using JMP 11.0 (SAS Institute, Cary, NC).

## Results

### Patient Characteristics

We enrolled total 11 HF patients who met the inclusion criteria. Among 11 patients, 2 patients did not use ASV > 4 hours per night. Therefore, 9 patients completed the study protocol, and we analyzed these 9 patients in the current study.

The underlying causes of HF were dilated cardiomyopathy in five patients (55.6%), dilated hypertrophic cardiomyopathy in one patient (11.1%), ischemic cardiomyopathy in one patient (11.1%), and others in two patients (22.2%). NYHA functional class was II in six patients (66.7%) and III in three patients (33.3%). Mean LVEF was 36.1 ± 5.3% ranging from 22% to 48%. Medical treatment included angiotensin-converting enzyme inhibitors or angiotensin receptor blockers in nine patients (100%), beta blockers in nine patients (100%), and diuretics in six patients (66.7%) (Table 1). Among the nine patients, six met the criteria for sleep apnea, with an apnea-hypopnea index (AHI) of greater than 15.^14^,^26^ Among these 6 patients, 5 patients were obstructive sleep apnea and one patient was central sleep apnea. Mean AHI was 22.3 ± 14.4/h, and mean 3% oxygen desaturation index was 20.3 ± 14.7/h at baseline. AHI means numbers of apnea and hypopnea per hour. AHI reflects the severity of sleep apnea disorders and AHI > 15 is defined as sleep apnea.^26^ AHI ranged from 15 to 30 is defined as moderate sleep apnea.^33^ The oxygen desaturation index (ODI) has been used to identify screening tools for sleep apnea. ODI is calculated as drop in oxygen saturation greater than equal 3% in comparison with immediately preceding basal value. We did not change medical therapy through the 6 months of this study.

**Table 1:** Baseline demographic and clinical characteristics of study patients (n = 9)

Characteristics	Value
Age (years)	57.3 ± 17.3
Sex (male/female)	6/3
BMI (kg/m^2^)	21.4 ± 5.5
NYHA classification (II/III)	6/3
Etiology of HF	
Dilated cardiomyopathy	5 (55.6%)
Dilated hypertrophic cardiomyopathy	1 (11.1%)
Ischemic cardiomyopathy	1 (11.1%)
Others	2 (22.2%)
Heart rate (beats/min)	79.8 ± 27.7
Systolic blood pressure (mmHg)	105.4 ± 22.0
Diastolic blood pressure (mmHg)	64.6 ± 16.4
Serum biochemical tests	
Creatinine (mg/dL)	0.92 ± 0.23
Log BNP (pg/mL)	2.48 ± 0.54
Echocardiography	
LVEF (%)	36.1 ± 5.3
LVDd (mm)	63.4 ± 11.8

Values are mean ± standard deviation or number (proportion, %)

*BMI*, body mass index; *BNP*, brain-type natriuretic peptide; *LVEF*, left ventricular ejection fraction; *LVDd*, left ventricular diastolic diameter

### ASV Treatments

Clinical characteristics at baseline and after 6 months of ASV treatment are shown in Table 2. Mean daily use of ASV was 4.8 ± 0.7 hours. AHI was significantly decreased (22.3 ± 14.4 to 1.9 ± 4.1/h; *P* = 0.040) and minimum peripheral oxygen saturation increased (83.1 ± 8.3 to 91.0 ± 3.3%; *P* = 0.016).

**Table 2:** Clinical characteristics at baseline and after 6 months of ASV Treatment (n = 9)

	Baseline	After ASV treatment	*P* value
AHI (/h)	18.8 (13.9-36.2)	1.0 (0.2-2.6)	0.004
Physical findings			
Heart rate (beats/min)	79.8 ± 27.7	67.8 ± 11.7	0.100
Systolic blood pressure (mmHg)	105.4 ± 22.0	100.7 ± 12.2	0.235
Diastolic blood pressure (mmHg)	64.7 ± 16.4	59.7 ± 8.8	0.203
Echocardiography			
LVEDV (mL)	149.1 (92.8-255.7)	86.1 (51.1-200.5)	0.164
LVESV (mL)	86.1 (51.2-200.5)	96.7 (52.9-186.7)	0.287
LVEF (%)	36.1 ± 5.3	39.2 ± 5.1	0.096
Biochemical tests			
Log BNP (pg/mL)	2.48 ± 0.54	2.07 ± 0.69	0.048
Plasma norepinephrine (pg/L)	345.0 (312.5-485.0)	360.0 (184.5-521.5)	0.496
Urinary norepinephrine (pg/mL)	112.1 (90.3-191.5)	135.0 (101.6-188.7)	0.367

Values are mean ± standard deviation or as median (interquartile range)

*ASV*, Adaptive servo-ventilation; *AHI*, apnea-hypopnea index; *LVEDV*, left ventricular end-diastolic volume; *LVESV*, left ventricular end-systolic volume

### Effects of ASV

There were no significant changes in heart rate and systolic or diastolic blood pressure (Table 2). LVEF tended to increase (36.1 ± 5.3 to 39.2 ± 5.1%; *P* = 0.096). LV end-diastolic volume (180.2 ± 95.2 to 160.9 ± 81.8 mL; *P* = 0.217) and LV end-systolic volume (122.3 ± 86.3 to 107.4 ± 72.4 mL; *P* = 0.272) tended to decrease after the ASV therapy. In addition, plasma and urinary norepinephrine concentrations did not change before and after ASV treatment (Table 2). The log plasma BNP concentration significantly decreased (*P* = 0.048) after ASV treatment.

### Effects of ASV on Cardiac SNF

**Figure 2:**
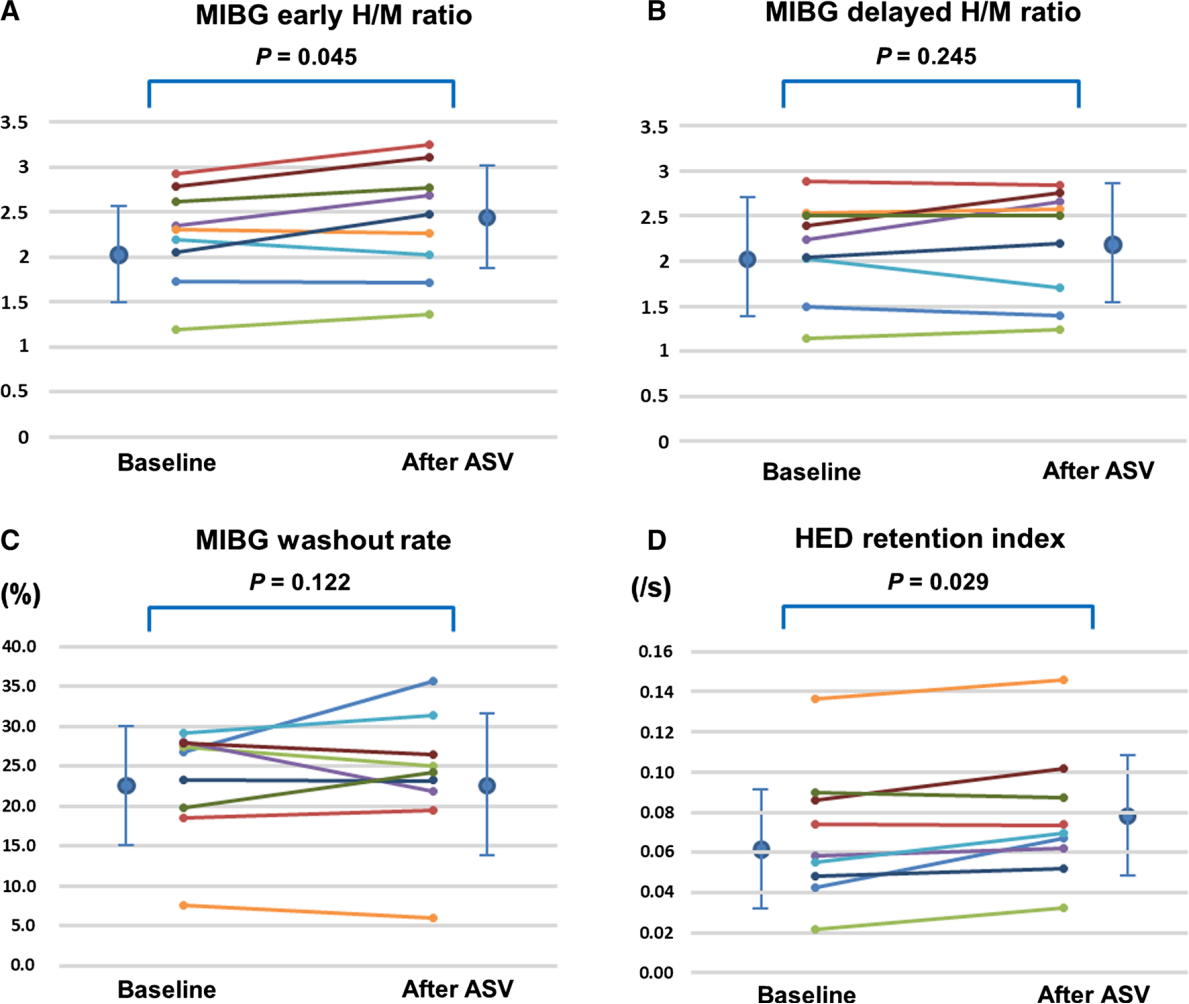
Changes in early H/M ratio, washout rate, and retention index after ASV treatment. **A** Early H/M ratio of ^123^I-MIBG before and after ASV treatment. H/M was estimated by standard approach using planar imaging^29^,^53^. **B** Delayed H/M ratio of ^123^I-MIBG before and after ASV treatment. **C** Washout rate of ^123^I-MIBG before and after ASV treatment. **D** Retention index of ^11^C-HED PET/CT before and after ASV treatment. All abbreviations are the same as in Figure 1. Mean ± SD of each parameter: Early H/M ratio of ^123^I-MIBG (baseline: 2.19 ± 0.58, after ASV: 2.40 ± 0.67), Delayed H/M ratio of ^123^I-MIBG (baseline: 2.07 ± 0.61, after ASV: 2.22 ± 0.66), Washout rate of ^123^I-MIBG (baseline: 23.8 ± 7.3%, after ASV: 23.8 ± 8.8%), Retention index of ^11^C-HED PET/CT (baseline: 0.068 ± 0.033/s, after ASV: 0.075 ± 0.034/s)

**Figure 3:**
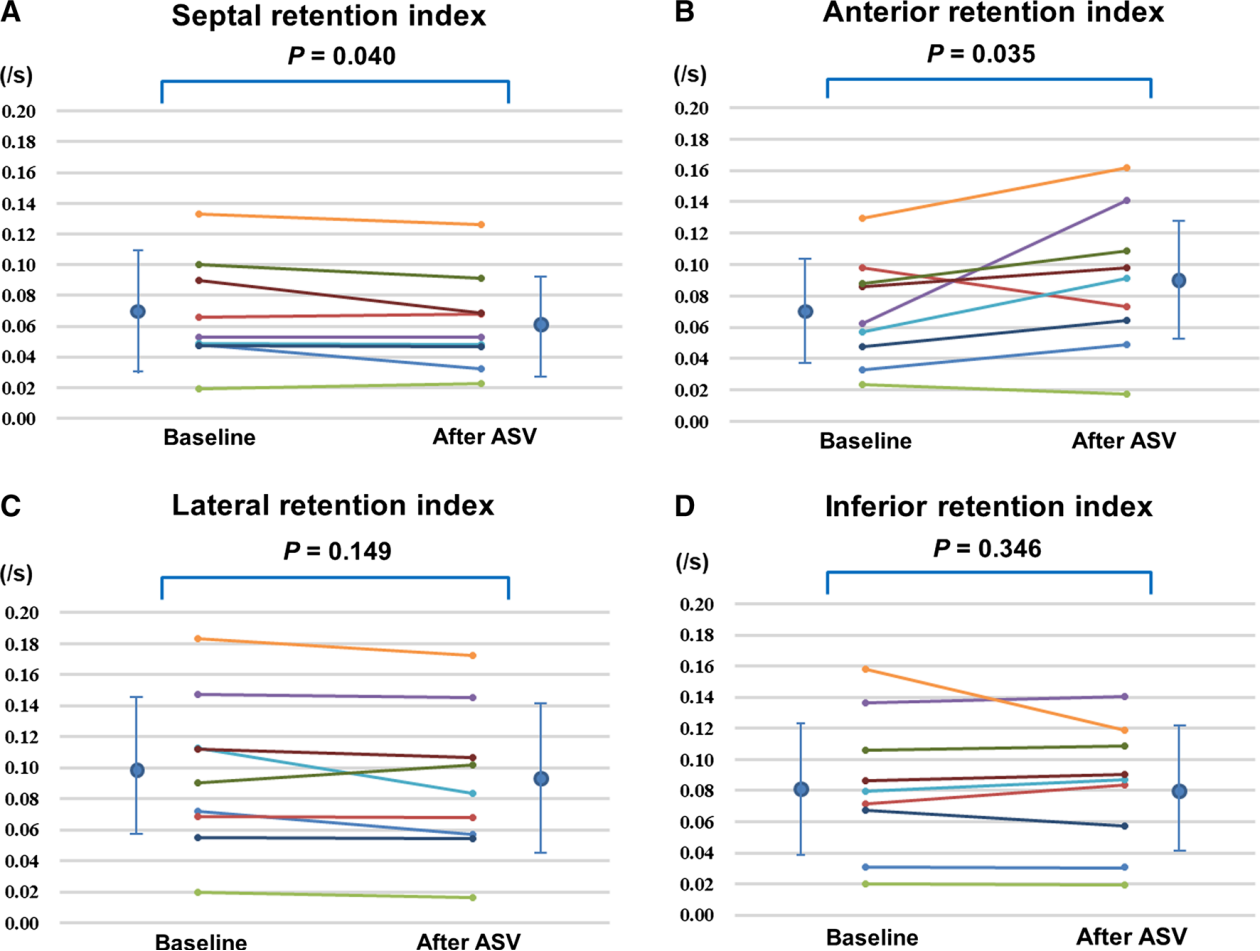
Change in regional retention index after ASV treatment. **A** Septal, **B** Anterior, **C** Lateral, **D** Inferior. *ASV*, adaptive servo-ventilation. Mean ± SD of each regional retention index: Septal (baseline: 0.067 ± 0.035/s, after ASV: 0.061 ± 0.032/s), Anterior (baseline: 0.070 ± 0.034/s, after ASV: 0.090 ± 0.045/s), Lateral (baseline: 0.096 ± 0.050/s, after ASV: 0.089 ± 0.048/s), Inferior (baseline: 0.084 ± 0.045/s, after ASV: 0.082 ± 0.040/s)

In ^123^I-MIBG scintigraphy, the early H/M ratio significantly increased (2.19 ± 0.58 to 2.40 ± 0.67; *P* = 0.045) (Figures 1 and 2). The delayed H/M ratio and washout rate did not change before and after 6 months of ASV treatment (delayed H/M ratio: 2.07 ± 0.61 to 2.22 ± 0.66; *P* = 0.245, washout rate: 23.8 ± 7.3% to 23.8 ± 8.8%; *P* = 0.122). The whole LV ^11^C-HED RI significantly increased (0.068 ± 0.033/s to 0.075 ± 0.034/s; *P* = 0.029) after ASV treatment. In addition, the RI significantly increased in the anterior region (0.070 ± 0.034/s to 0.090 ± 0.045/s; *P* = 0.035), and decreased in the septal region (0.067 ± 0.035/s to 0.061 ± 0.032/s; *P* = 0.040) (Figure 3). There were no significant changes in the RI in the inferior or lateral regions.

## Discussion

ASV treatment for 6 months improved AHI and plasma BNP levels. It also improved global LV and anterior presynaptic function but did not change the ^123^I-MIBG imaging washout rate in HF patients.

### Effects of ASV

Apnea-hypopnea index (AHI) means numbers of apnea and hypopnea per hour. AHI reflects the severity of sleep apnea disorders and AHI > 15 is defined as sleep apnea.^26^ Based on the American Academy of Sleep Medicine (AASM) criteria, AHI ranged from 15 to 30 is defined as moderate sleep apnea.^33^ In the current study, mean AHI was 22.3 ± 14.4/h. Although 3 patients were not defined as sleep apnea, the patients having sleep apnea may have been categorized as moderate severity. ASV treatment decreased the AHI in agreement with results of previous studies.^34^,^35^ The average use of ASV was 4.8 ± 0.7 h per night, showing good compliance of ASV during the study period. Sufficient use of ASV per night likely contributed to the decrease in AHI. Although there was no significant different, LV remodeling parameters such as LVEDV and LVESV decreased 6 months after the ASV treatment. Previous study by Koyama et al. showed significant increase in LVEF and significant decrease in LVEDV and LVESV.^13^ The tendency of ASV treatment effects on LVEF and LV remodeling parameters after the ASV treatments of the current study agree with previous study by Koyama et al. In terms of severity of sleep disorder, the previous study by Koyama enrolled more severe sleep apnea patients (AHI 35.7 ± 13.7/h) than that of the current study (22.3 ± 14.4/h). The difference of patients background may have had impacts on the differences between the current study and previous study by Koyama et al. This difference may have also had impacts on the neurohormonal parameter changes. In addition, log BNP significantly decreased after 6 months of ASV therapy. Though there are some differences, overall these results agree with those of previous studies using ASV in HF.^13^,^36^

### Presynaptic Function and Washout Parameter

Among recent applications of diagnostic, imaging is the non-invasive evaluation of cardiac SNF in HF patients. In particular, both ^123^I-MIBG imaging and ^11^C-HED PET are useful modalities for non-invasive evaluation for cardiac SNF.^18^ It has been reported that the evaluation of cardiac SNF through ^123^I-MIBG imaging can be used to stratify risk in patients with HF. The low H/M ratio as determined through ^123^I-MIBG planar images can predict sudden cardiac death in HF regardless of LVEF.^37^ Optimal medical therapy and cardiac resynchronization therapy have also been reported to improve cardiac SNF, as evaluated by ^123^I-MIBG imaging, and to reduce mortality in HF patients.^38^ In a recent ^123^I-MIBG imaging study, Koyama et al reported that ASV therapy ameliorated SNF in HF patients.^13^ It appears that ^123^I-MIBG imaging is a reliable diagnostic tool for evaluating cardiac SNF and prognosis in HF. In those studies, most investigators have used planar cardiac images and made semi-quantitative assessment of global LV sympathetic nervous function such as the H/M ratio and washout rate.^39^^–^41

^11^C-HED PET/CT has emerged as a new modality to evaluate cardiac SNF. It has a high spatial resolution and can also be used to accurately quantify cardiac SNF at both the LV global and regional levels. In particular, the RI in ^11^C-HED PET has been closely correlated with cardiovascular events in HF.^17^ These accurate quantitative parameters are a major advantage of ^11^C-HED PET/CT

As the physical short half-life of ^11^C-HED is 20 min, ^11^C-HED cannot provide washout parameters as ^123^I-MIBG imaging can.^25^ The ideal SNF measurements would be precise quantifications with uptake as well as washout parameters. No imaging tool has been established to achieve this goal. In this regard, we evaluated the effects of ASV on SNF using ^11^C-HED PET as a quantitative marker of presynaptic function, and ^123^I-MIBG imaging as a washout parameter in the current study regarding to previous studies.^23^,^42^^–^44 This is a relatively new concept for SNF measurements applied for therapeutic effects of ASV, which takes the previous study by Koyama et al.^13^ further.

We found that the global ^11^C-HED PET/CT RI improved after 6 months of ASV therapy. The early H/M ratio also significantly increased, a finding that aligns with those of a previous study.^13^ In addition to performing semi-quantitative H/M ratio measurement using ^123^I-MIBG imaging, we confirmed previous reports using precise quantitative assessment using ^11^C-HED PET/CT. Reduced AHI from using ASV may have contributed to improving presynaptic sympathetic nervous function. In contrast, the delayed H/M ratio and washout rate did not change in ^123^I-MIBG imaging. The reasons for this finding are unclear. It is possible that improvements in ^123^I-MIBG washout appear later than do improvements in presynaptic function via uptake-1. This means that both global ^11^C-HED PET RI and the early H/M ratio determined by ^123^I-MIBG may indicate improvements of presynaptic cardiac SNF during the early phase of ASV therapy in HF patients. Long-term observational studies are required to confirm this hypothesis. The early H/M did not reach statistically significant improvement but the mean value was improved after the ASV therapy in the current study. Thus, another possible explanation for this discrepancy could be the small sample size of the current study

### Regional Sympathetic Nervous Function

In the current study, ASV therapy improved cardiac sympathetic function in the anterior LV region as well as global LV SNF based on ^11^C-HED PET/CT data. This finding provides additional insights beyond those of the previous study by Koyama et al.^13^ Improvement of cardiac SNF in the anterior LV may be associated with innervation within the heart. Regional patterns of myocardial sympathetic denervation have been observed in patients with dilated cardiomyopathy, diabetic autonomic neuropathy, and prior myocardial infarction with SNF measured by ^11^C-HED PET, with the lowest RI reported in the inferoapical region.^17^,^45^,^46^ In animal studies, the anterior region of the LV was found to be the most densely innervated with sympathetic fibers, and the concentration of catecholamine was the highest at the anterior and basal LV walls.^47^,^48^ We found the beneficial effects of ASV therapy on cardiac SNF in the anterior region. The denser sympathetic innervation of this area may be the mechanism behind the changes in cardiac SNF with ASV therapy, which lead to easier detection by ^11^C-HED PET/CT. In contrast, among 9 cases, 4 cases actually reduced HED RI in septum. In HF patients, increased LV end-diastolic pressure and associated pulmonary edema will cause pulmonary hypertension, and the septum is stretched to become dysfunctional by making the helix fibers more transverse. Furthermore, as the left ventricle becomes dilate, the septum will encroach on the RV cavity in the chronic period. Therefore, the left ventricular septum could be more susceptible to damage than other left ventricular wall due to the left ventricular pressure and volume loading.^49^ These specific function and structural elements of the septum may be closely related to SNF functional change. However, the magnitude of improving anterior RI was bigger than that of RI reduction in septum (31.4 ± 46.1% vs −6.4 ± 14.5%). As a result, whole LV sympathetic nervous activity improved after the ASV therapy in the current study.

### Study Limitations

This study was a single-center study with a small sample size. While the number of patients was small, PET measurements can provide segment-by-segment assessment of absolute RI. When combined with a repeated measures analysis, in which each myocardial segment within each patient can serve as its own control, the study design may provide a valid assessment of the effects of ASV therapy despite the limited sample size. A similar sample size was used in previous studies evaluating sympathetic nervous function changes after therapeutic interventions using ^11^C HED PET.^50^,^51^ Despite the relatively small sample size, the change in sympathetic nervous function after the ASV measurements by ^11^C HED PET and ^123^I MIBG scintigraphy was demonstrated. However, the small sample size may have had impacts on the data with early H/M and washout rate. In this regard, a larger population is required to confirm the results. The extent to which improvements in cardiac SNF influence prognosis in patients with HF treated with ASV is not known. Cowie et al. reported that ASV therapy did not influence mortality in patients with chronic HF and central sleep apnea.^52^ However, a close look at the study population reveals that there are some significant differences between the SERVE-HF trial, the current study and the previous study by Koyama et al.^13^ The SERVE-HF trial seems to include end-stage HF patients such as those in NYHA functional class 4. Second, over 50% of the enrolled population in the SERVE-HF trial had HF due to coronary artery disease. In the study by Koyama et al. and the current study, most of the patients had NYHA class 2 HF and mildly to moderately reduced LVEF, possibly indicating that these populations may still have viable myocardium that can respond to specific new or additional treatment including ASV. In contrast, the study population in the SERVE-HF trial seemed not to have sufficient viable myocardium that could respond to the additional treatment. In addition, patients with HF due to CAD may have extensive scarring which may also contribute to a diminished response to new treatments. Despite these differences, ASV may still be able to improve cardiac function in patients with mild-to-moderate HF not caused by CAD. In this regard, improvement to cardiac sympathetic nervous function may be one of the key factors for therapeutic effects. The study populations in the current study and in the previous study by Koyama et al. are small. Therefore, as a next step, we would definitely need a larger clinical trials to test this hypothesis. Our findings suggest that ASV improves cardiac SNF in HF patients, but we cannot draw any conclusions about their long-term prognosis.

## Conclusions

ASV reduced AHI in HF patients. Early ^123^I-MIBG H/M ratio and ^11^C-HED RI significantly improved, whereas ^123^I-MIBG washout ratio did not change with ASV. A comprehensive approach using a combination of ^11^C-HED PET and ^123^I-MIBG imaging could be used to evaluate cardiac SNF and provide regional and global SNF uptake parameters and global washout parameters in HF patients. ASV therapy may mediate its beneficial effects in HF by initially improving presynaptic SNF.

## New knowledge gained

The combined use of ^11^C-HED PET and ^123^I-MIBG imaging may play a role in the detection of presynaptic cardiac sympathetic nervous function for the evaluation of new heart failure therapy.

## Electronic supplementary material

Below is the link to the electronic supplementary material.

Supplementary material 1 (PPTX 2292 kb)

## Abbreviations

ASV: Adaptive servo-ventilation
BNP: Brain-type natriuretic peptide
HED: ^11^C-hydroxyephedrine
HF: Heart failure
LVEF: Left ventricular ejection fraction
MIBG: ^123^I-metaiodobenzylguanidine
PET: Positron emission tomography
RI: Retention index
SNF: Sympathetic nervous function
SPECT: Single-photon emission computed tomography
