# Supplementary material for: Early therapeutic effects of adaptive servo-ventilation on cardiac sympathetic nervous function in patients with heart failure evaluated using a combination of 11C-HED PET and 123I-MIBG SPECT
Source: J Nucl Cardiol. 2017 Nov 27;26(4):1079–89. doi: 10.1007/s12350-017-1132-4 (PMC6660491; doi:10.1007/s12350-017-1132-4)
Supplement: Supplementary file 1 — Supplementary material 1 (PPTX 2292 kb) [file 12350_2017_1132_MOESM1_ESM.pptx]

## Slide 1
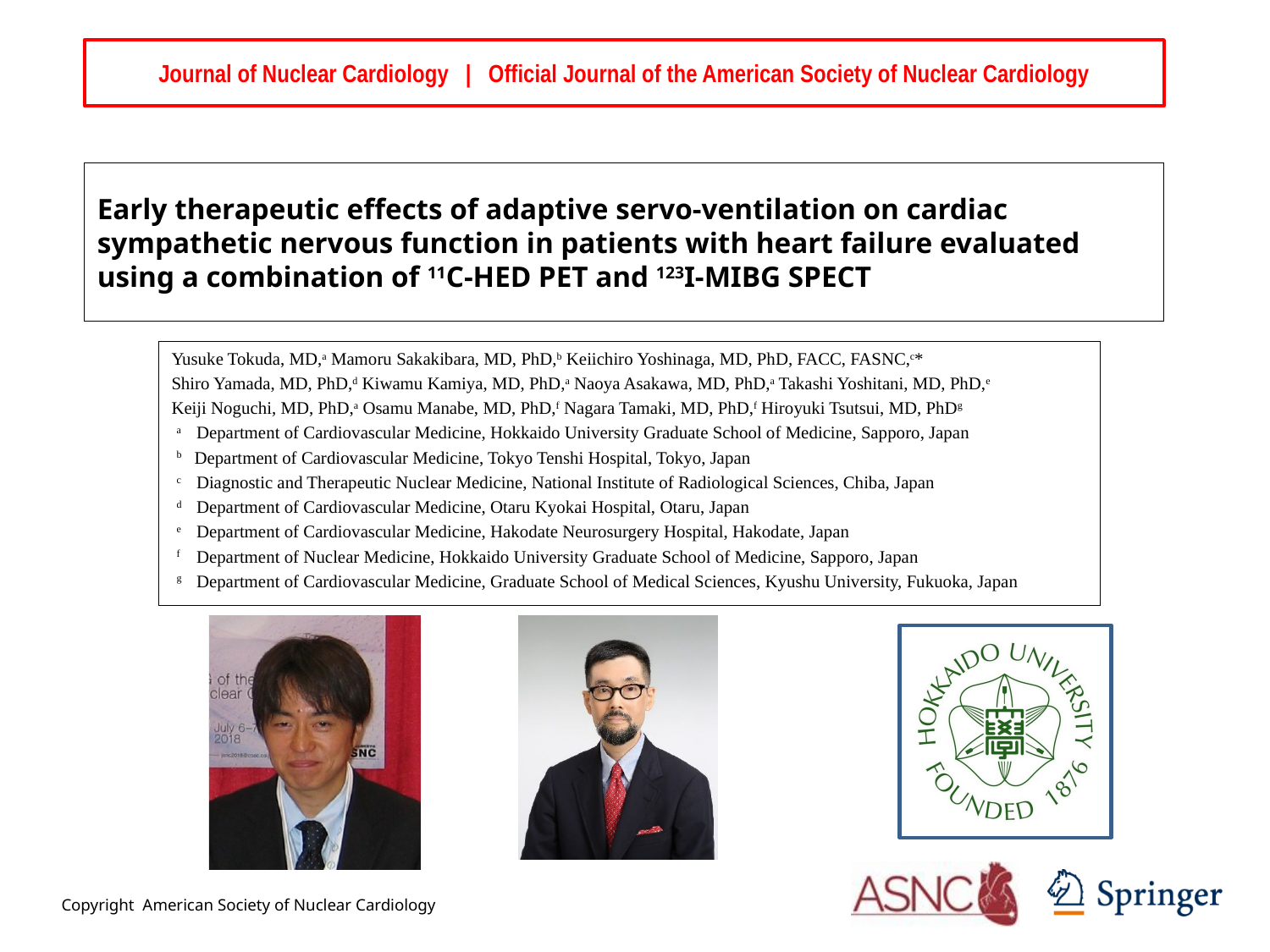

Journal of Nuclear Cardiology | Official Journal of the American Society of Nuclear Cardiology
# Early therapeutic effects of adaptive servo‐ventilation on cardiac sympathetic nervous function in patients with heart failure evaluated using a combination of 11C-HED PET and 123I-MIBG SPECT
Yusuke Tokuda, MD,a Mamoru Sakakibara, MD, PhD,b Keiichiro Yoshinaga, MD, PhD, FACC, FASNC,c*
Shiro Yamada, MD, PhD,d Kiwamu Kamiya, MD, PhD,a Naoya Asakawa, MD, PhD,a Takashi Yoshitani, MD, PhD,e
Keiji Noguchi, MD, PhD,a Osamu Manabe, MD, PhD,f Nagara Tamaki, MD, PhD,f Hiroyuki Tsutsui, MD, PhDg
 a	Department of Cardiovascular Medicine, Hokkaido University Graduate School of Medicine, Sapporo, Japan
 b Department of Cardiovascular Medicine, Tokyo Tenshi Hospital, Tokyo, Japan
 c	Diagnostic and Therapeutic Nuclear Medicine, National Institute of Radiological Sciences, Chiba, Japan
 d	Department of Cardiovascular Medicine, Otaru Kyokai Hospital, Otaru, Japan
 e	Department of Cardiovascular Medicine, Hakodate Neurosurgery Hospital, Hakodate, Japan
 f	Department of Nuclear Medicine, Hokkaido University Graduate School of Medicine, Sapporo, Japan
 g	Department of Cardiovascular Medicine, Graduate School of Medical Sciences, Kyushu University, Fukuoka, Japan
Copyright American Society of Nuclear Cardiology

## Slide 2
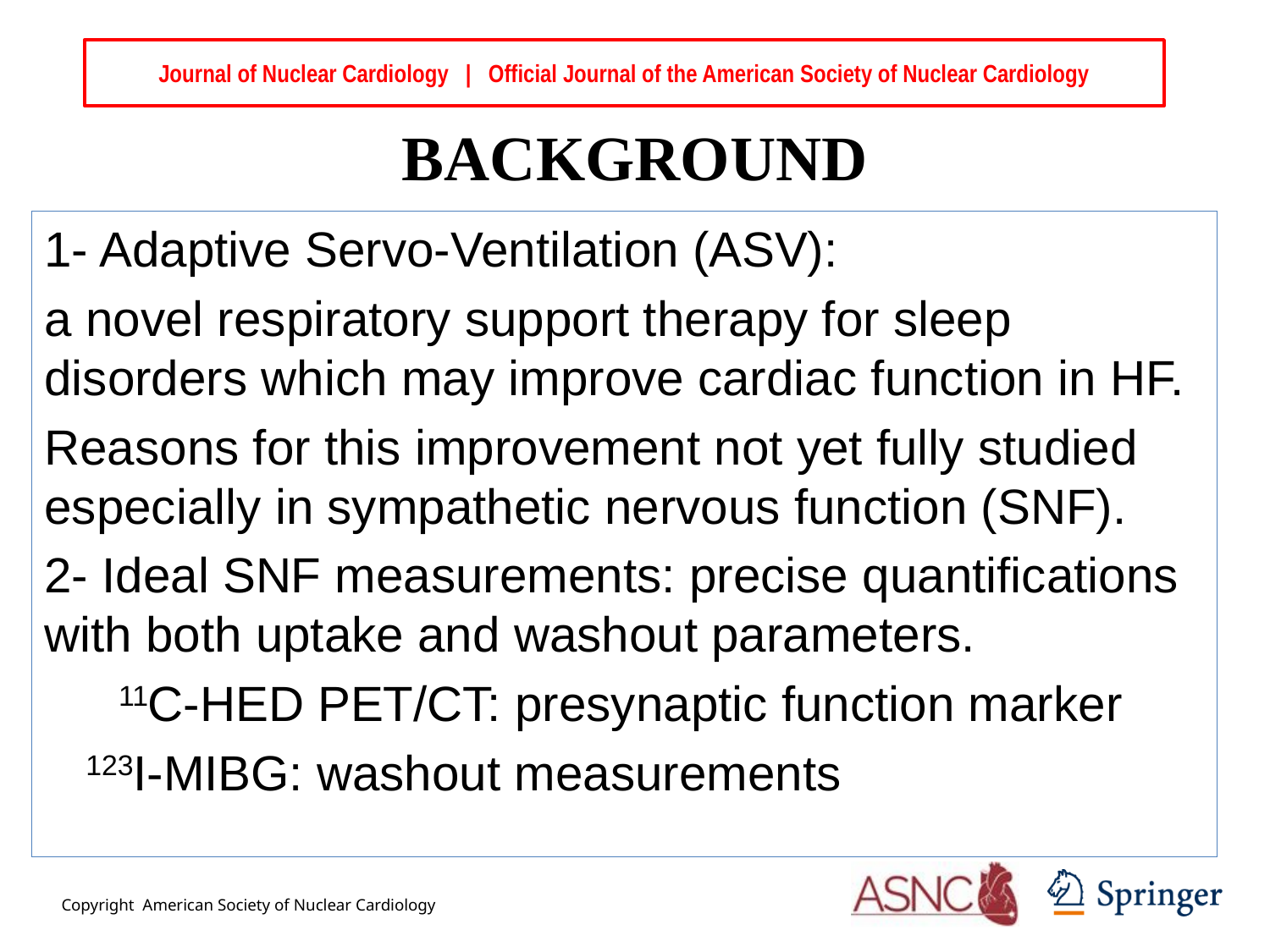

Journal of Nuclear Cardiology | Official Journal of the American Society of Nuclear Cardiology
# BACKGROUND
1- Adaptive Servo-Ventilation (ASV):
a novel respiratory support therapy for sleep disorders which may improve cardiac function in HF.
Reasons for this improvement not yet fully studied especially in sympathetic nervous function (SNF).
2- Ideal SNF measurements: precise quantifications with both uptake and washout parameters.
　 11C-HED PET/CT: presynaptic function marker
 123I-MIBG: washout measurements
Copyright American Society of Nuclear Cardiology

## Slide 3
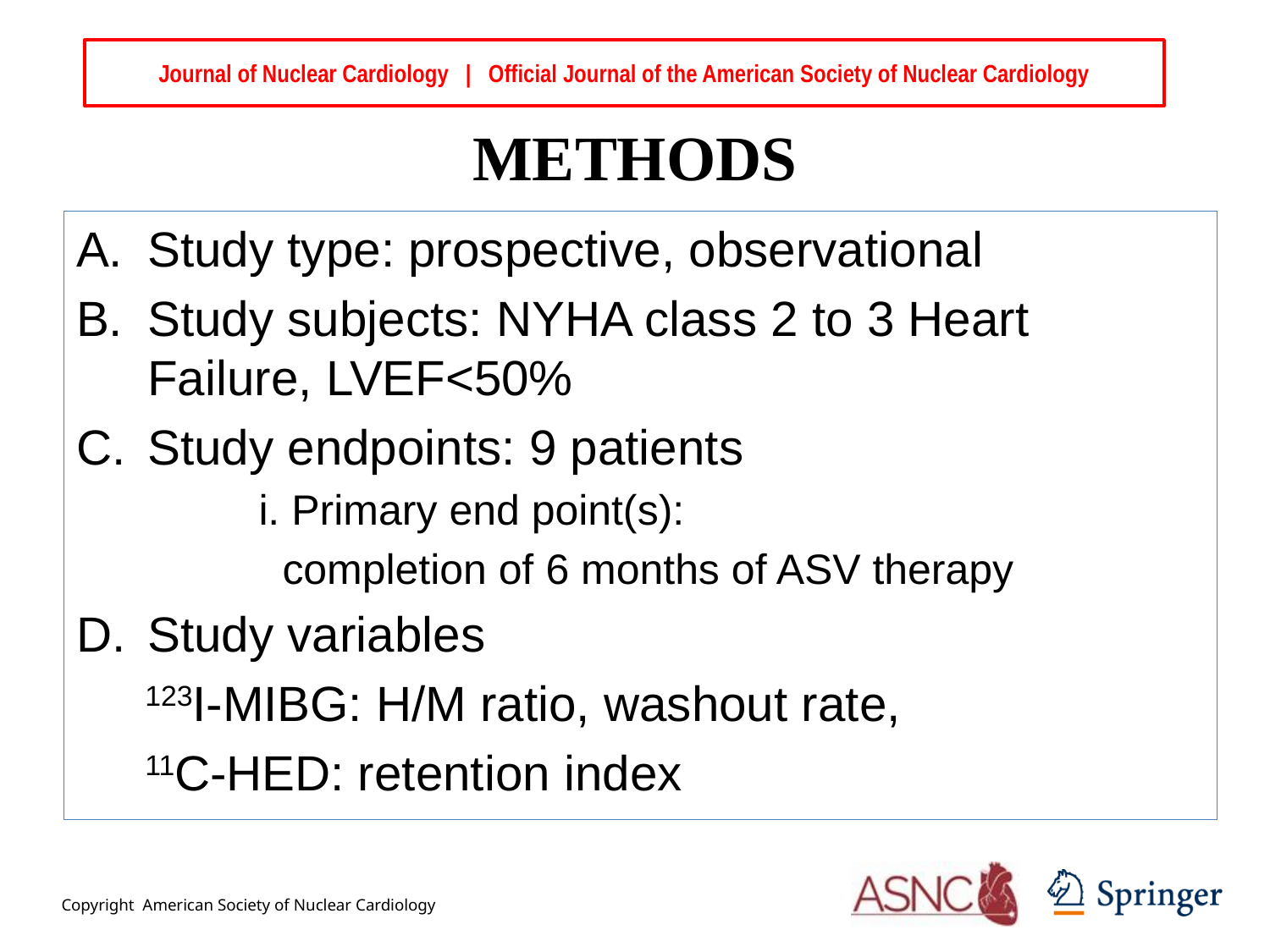

Journal of Nuclear Cardiology | Official Journal of the American Society of Nuclear Cardiology
# METHODS
Study type: prospective, observational
Study subjects: NYHA class 2 to 3 Heart Failure, LVEF<50%
Study endpoints: 9 patients
	i. Primary end point(s):
	 completion of 6 months of ASV therapy
Study variables
 123I-MIBG: H/M ratio, washout rate,
 11C-HED: retention index
Copyright American Society of Nuclear Cardiology

## Slide 4
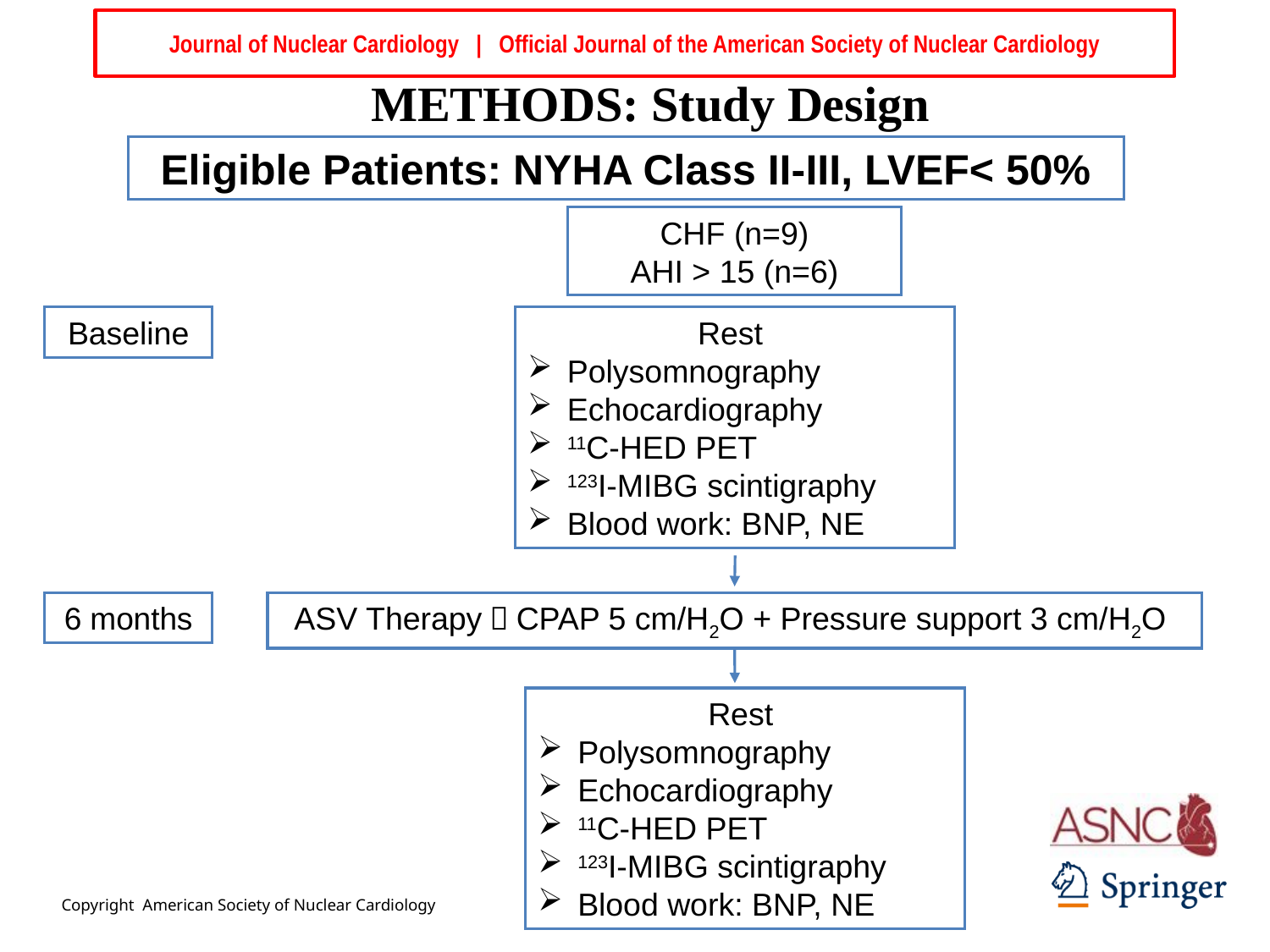

Journal of Nuclear Cardiology | Official Journal of the American Society of Nuclear Cardiology
# METHODS: Study Design
Eligible Patients: NYHA Class II-III, LVEF< 50%
CHF (n=9)
AHI > 15 (n=6)
Baseline
Rest
Polysomnography
Echocardiography
11C-HED PET
123I-MIBG scintigraphy
Blood work: BNP, NE
6 months
ASV Therapy：CPAP 5 cm/H2O + Pressure support 3 cm/H2O
Rest
Polysomnography
Echocardiography
11C-HED PET
123I-MIBG scintigraphy
Blood work: BNP, NE
Copyright American Society of Nuclear Cardiology

## Slide 5
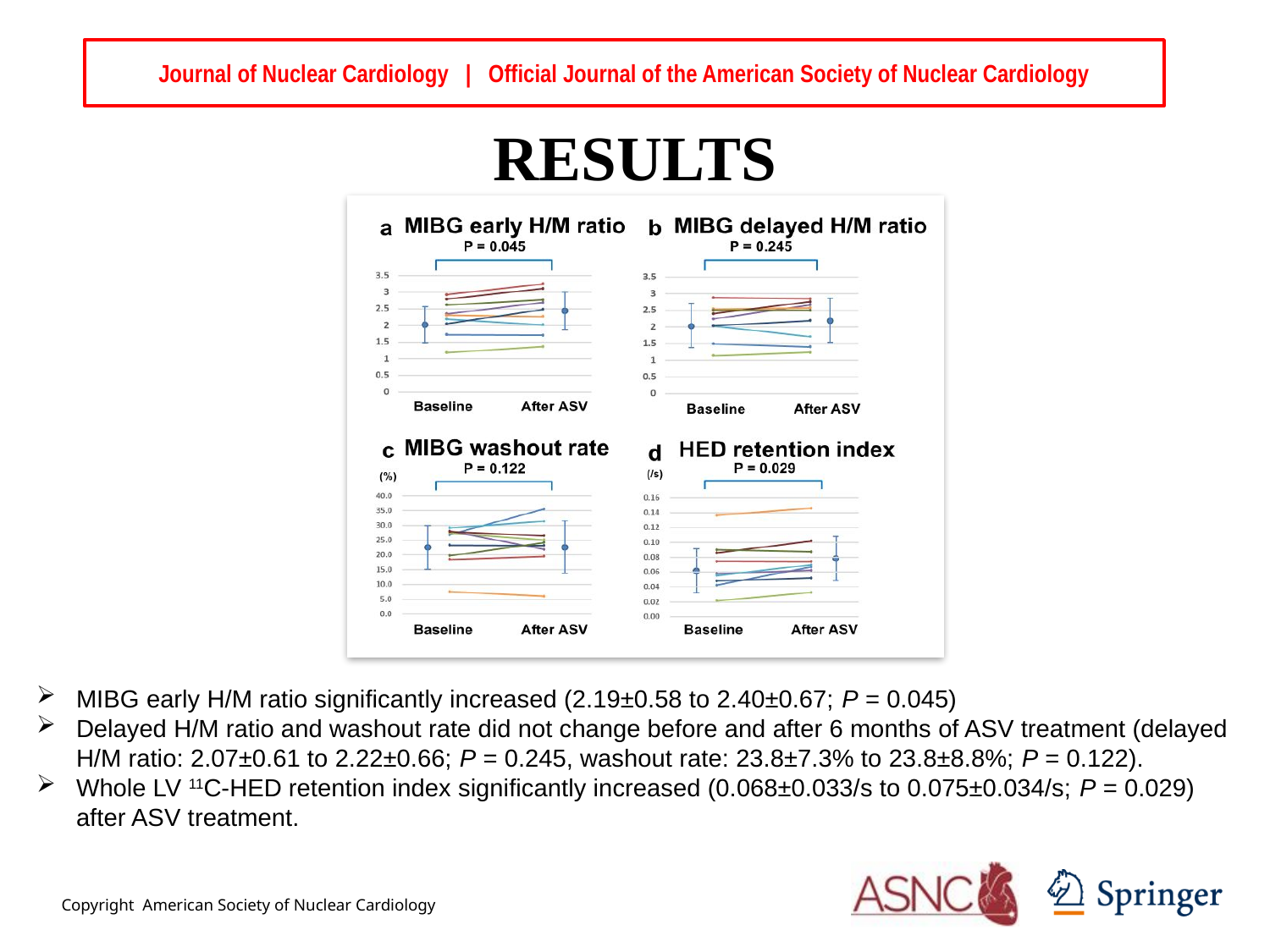

Journal of Nuclear Cardiology | Official Journal of the American Society of Nuclear Cardiology
# RESULTS
MIBG early H/M ratio significantly increased (2.19±0.58 to 2.40±0.67; P = 0.045)
Delayed H/M ratio and washout rate did not change before and after 6 months of ASV treatment (delayed H/M ratio: 2.07±0.61 to 2.22±0.66; P = 0.245, washout rate: 23.8±7.3% to 23.8±8.8%; P = 0.122).
Whole LV 11C-HED retention index significantly increased (0.068±0.033/s to 0.075±0.034/s; P = 0.029) after ASV treatment.
Copyright American Society of Nuclear Cardiology

## Slide 6
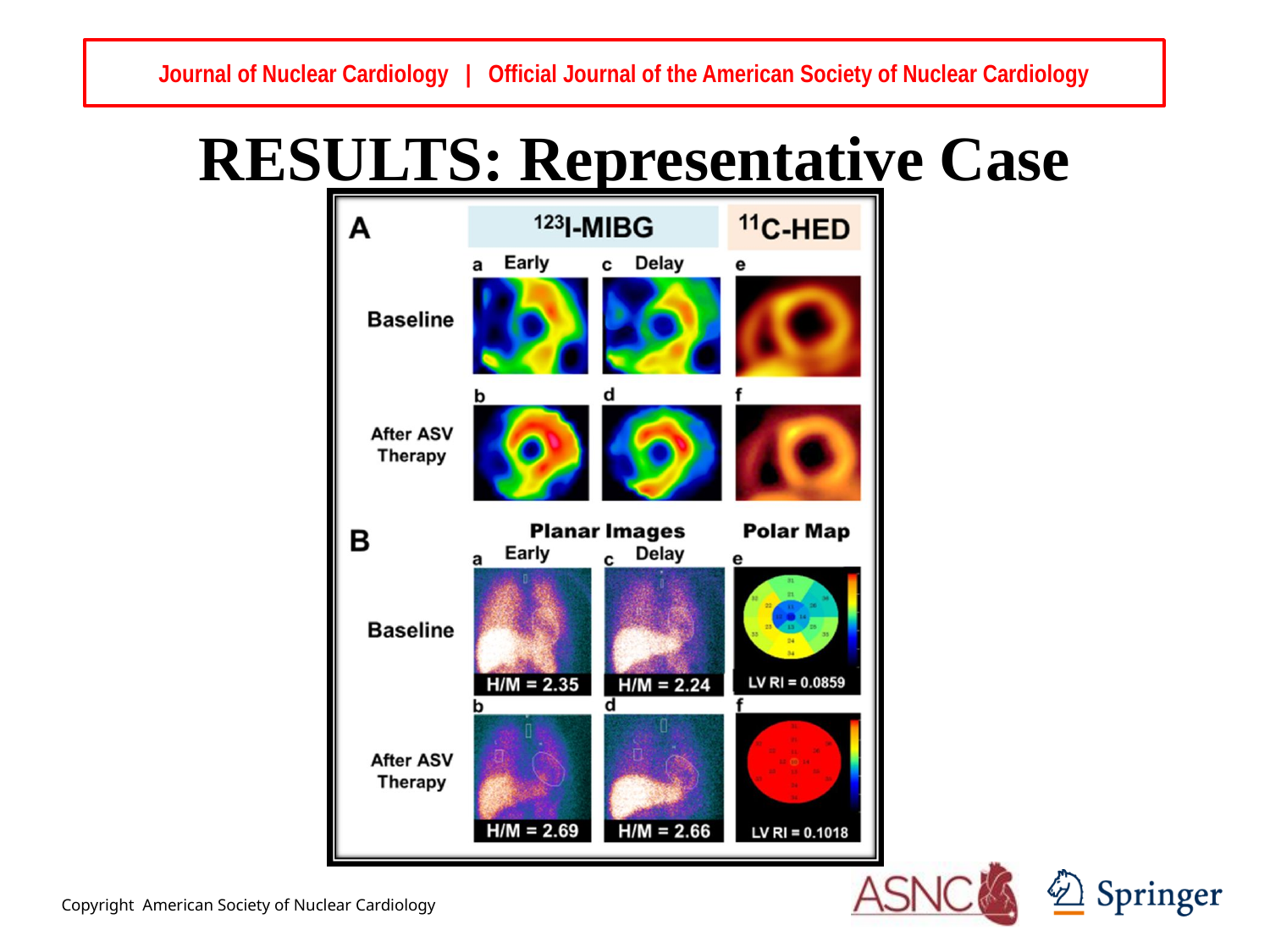

Journal of Nuclear Cardiology | Official Journal of the American Society of Nuclear Cardiology
# RESULTS: Representative Case
Copyright American Society of Nuclear Cardiology

## Slide 7
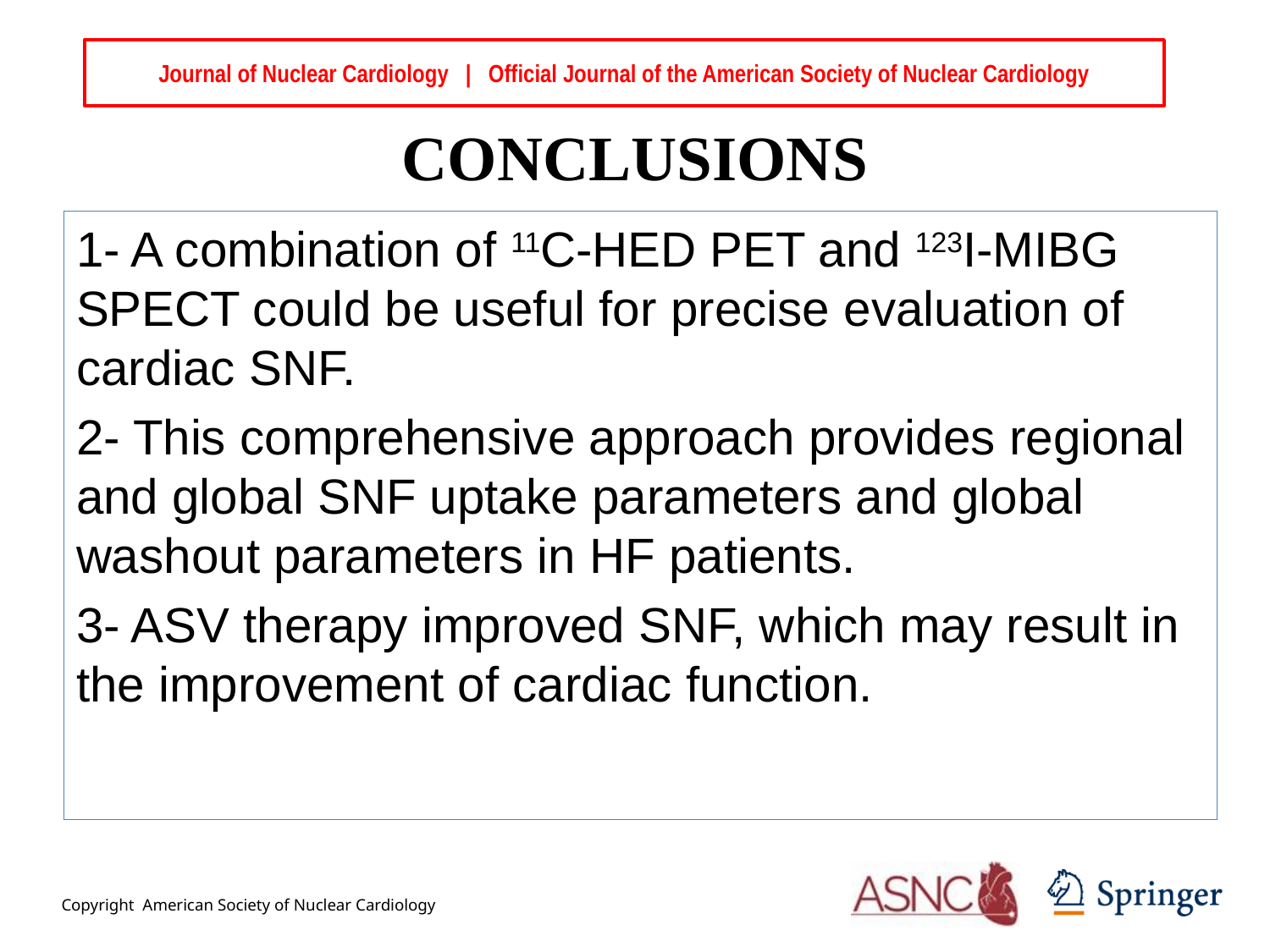

Journal of Nuclear Cardiology | Official Journal of the American Society of Nuclear Cardiology
# CONCLUSIONS
1- A combination of 11C-HED PET and 123I-MIBG SPECT could be useful for precise evaluation of cardiac SNF.
2- This comprehensive approach provides regional and global SNF uptake parameters and global washout parameters in HF patients.
3- ASV therapy improved SNF, which may result in the improvement of cardiac function.
Copyright American Society of Nuclear Cardiology
